# How People with Multiple Sclerosis Rate Their Quality of Life: An EQ-5D Survey via the UK MS Register

**DOI:** 10.1371/journal.pone.0065640

**Published:** 2013-06-11

**Authors:** Kerina H. Jones, David V. Ford, Philip A. Jones, Ann John, Rodden M. Middleton, Hazel Lockhart-Jones, Jeffrey Peng, Lisa A. Osborne, J. Gareth Noble

**Affiliations:** 1 College of Medicine, Swansea University, Swansea, Wales, United Kingdom; 2 Long Term & Chronic Conditions Centre, College of Human and Health Sciences, Swansea University, Swansea, Wales, United Kingdom; Innsbruck Medical University, Austria

## Abstract

**Introduction:**

The EQ-5D is a widely-used, standardised, quality of life measure producing health profiles, indices and states. The aims of this study were to assess the role of various factors in how people with Multiple Sclerosis rate their quality of life, based on responses to the EQ-5D received via the web portal of the UK MS Register.

**Methods:**

The 4516 responses to the EQ-5D (between May 2011 and April 2012) were collated with basic demographic and descriptive MS data and the resulting dataset was analysed in SPSS (v.20).

**Results:**

The mean health state for people with MS was 59.73 (SD 22.4, median 61), compared to the UK population mean of 82.48 (which is approximately 1SD above the cohort mean). The characteristics of respondents with high health states (at or above +1SD) were: better health profiles (most predictive dimension: Usual Activities), higher health indices, younger age, shorter durations of MS, female gender, relapsing-remitting MS, higher educational attainment and being in paid employment (all *p*-values<0.001). Conversely, the characteristics of respondents with low health states (at or below -1SD) were: poorer health profiles (most predictive dimension: Mobility), lower health indices, older age, longer durations of MS, male gender, progressive MS, lower educational attainment and having an employment status of sick/disabled (*p* = 0.0014 for age, all other *p*-values<0.001). Particular living arrangements were not associated with either the high or low health status groups.

**Conclusions:**

This large-scale study has enabled in-depth analyses on how people with MS rate their quality of life, and it provides new knowledge on the various factors that contribute to their self-assessed health status. These findings demonstrate the impact of MS on quality of life, and they can be used to inform care provision and further research, to work towards enhancing the quality of life of people with MS.

## Introduction

The EQ-5D is a widely used, standardised, health-related quality of life measure developed by the EuroQol Group to provide a simple generic assessment for use in clinical and economic studies [1], [2]. It consists of the EQ-5D descriptive system and the EQ visual analogue scale (EQ-VAS). The descriptive system has five dimensions: Mobility, Self-care, Usual activities, Pain/discomfort and Anxiety/depression. Each dimension has 3 levels: no problems, some problems or severe problems, and this provides a health profile of the respondents. By taking the five digit responses, the health profiles can be translated into weighted health indices, which in the UK is usually done using the Time Trade Off (TTO) procedure [1], [3]. The EQ-VAS allows respondents to indicate their self-assessed health state on a visual analogue scale with 0 being their worst imagined health state, and 100 the highest. We will refer to these values as health profiles, health indices and health states/status in this study on quality of life in people with Multiple Sclerosis (MS) via the UK MS Register.

MS is a chronic, inflammatory, auto-immune condition characterised by demyelination of nerve fibres resulting in a range of symptoms and disabilities. There are estimated to be between 85,000 and 100,000 people with MS in the UK, but there is an acknowledged lack of epidemiological information about this debilitating condition [4]. The UK MS Register has been developed to address the need for a greater knowledge-base about MS. It has been designed to capture and bring together datasets from three main sources: clinical information systems operating in NHS Neurology clinics, routinely-collected administrative data, and directly from people with MS via a purpose built web portal operating as a questionnaire delivery platform [5]. The web portal was launched in May 2011 and, by April 2012, 8736 people with MS had enrolled, with further data collection underway as a continual process. The acquisition of data from Neurology clinics and administrative sources is progressing, subject to participant consent and data availability. The data model of the UK MS Register is innovative in its design and provides new opportunities for studying MS via linked data. The Register is based on the proven technologies and robust Information Governance arrangements in place in the Secure Anonymised Information Linkage (SAIL) system developed by the Health Information Research Unit (HIRU) [6], [7].

The web portal hosts a variety of validated questionnaires to capture information on the health and well-being of people with MS. These include the Hospital Anxiety and Depression Scale (HADS) [8], and the MS Disease Impact Scale-29 (MSIS-29) [9], and we have previously shown how the responses to these scales can be used to address research questions about the experience of living with MS [10], [11]. The EQ-5D is used internationally in general populations and condition-specific cohorts [12], [13] and because of its importance and wide usage, we included it as a questionnaire on the web portal of the UK Multiple Sclerosis Register.

### Research Aim

The aim of this study was to use the responses to the EQ-5D gained via the web portal to assess the role of various factors in how people with MS rate their quality of life. This is the largest known study of its kind in people with MS, and the first to use the EQ-5D responses from the UK MS Register. We therefore set out to describe the cohort for comparison with other studies and for those who may wish to use the Register data in the future. We compared the health status of our respondents with the general UK population and then examined the factors that characterise those with high self-rated health states and those with low self-rated health states. From previous studies, including those using the web portal data [10], [11], it was be expected that people with MS with high health states would differ in at least some of their characteristics compared to people with MS with low health states. For example, people with a progressive type of MS and longer durations of the condition would be likely to report poorer quality of life than those with relapsing-remitting MS and shorter durations. However, the influences of various other factors, such as which (if any) of the EQ-5D dimensions are most predictive of either a high of low self-rated health status, were not known.

## Methods

### Research Ethics and Governance

The UK MS Register study was peer-reviewed via the MS Society and has obtained ethical approval from the South West – Central Bristol Research Ethics Committee (11/SW/0160) as a research database [14]. Under this ethical approval, data collected via the web portal, the Neurology clinics and routine administrative sources can be anonymously linked using the SAIL methodologies provided that agreement to the portal terms of service (via the portal) and written informed participant consent (at the clinics) have been obtained. The working UK MS Register contains only anonymous data but facilities are in place to re-contact participants to take part in further research [15]. In future, the Register data will be made accessible for analysis by researchers external to the team, subject to regulatory and governance requirements. The final operating model for these arrangements is yet to be determined, but we are able at accommodate researcher requests to view the data in the interim, subject to any necessary amendments to regulatory and governance approvals and a non-disclosure agreement.

### Data Collection and Analysis

Adults in the UK who have MS have been able to enrol on the UK MS Register via the web portal since its launch in May 2011. From then until April 2012, 4516 people completed the EQ-5D questionnaire. These responses were collated with basic demographic, descriptive MS data and information on employment status, educational attainment and living arrangements from the questionnaire entitled ‘You, your MS and lifestyle’ [5] and the resulting dataset was analysed in SPSS (v.20). The health profiles were treated as categorical data, the health indices (calculated using the TTO method) [3] and health states were treated as continuous. All the continuous variables were assessed for normality using the Kolmogorov-Smirnov test and all were found to deviate significantly from the normal distribution (all *p* values 0.001). Because of this, non-parametric inferential tests were used: Spearman’s rank correlation coefficient to measure relationships between variables, the Mann-Whitney U test was used to assess differences between two independent samples, and the Kruskal-Wallis one-way analysis of variance was used to compare more than two independent samples. Logistic regression was used to predict the outcome of categorical variables, and the chi squared test was used to assess goodness of fit between categorical variables. A Bonferroni correction was used, where relevant, to avoid over-reporting significance where multiple tests were conducted. This was calculated as the significance level divided by the number of tests undertaken. In order to assess the characteristics associated with high health states the cohort was divided into two categories: those with a health status at or above 1SD above the mean (denoted as High-VAS), and those with a health status below this value. A similar method was used to study low health states, with the Low-VAS group denoted as having a health status at or below 1SD below the mean for the cohort. Workforce age is defined as < = 59 for women and < = 64 for men [16].

## Results

### Description of Respondents

The sample was comprised of 71.1% women and 28.9% men, representing a ratio of 2.46 women: 1 man (*N* = 4516). The distribution of types of MS were: 14.8% primary progressive MS (PPMS), 62.1% relapsing-remitting MS (RRMS), 8.1% secondary progressive MS (SPMS) and 14.9% did not know their type of MS (DKMS) (*N* = 4422). The mean age of the respondents was 50.7 years (SE 0.17, SD 11.2) with a median of 51 years (IQR 16). The mean time since diagnosis (by a Neurologist) was 10.9 years (SE 0.15, SD 8.9) with a median of 9 years (IQR 12). The mean health index was 0.567 (SE 0.003, SD 0.207) with a median of 0.596 (IQR 0.242). The mean health state was 59.73 (SE 0.33, SD 22.42) with a median of 61 (IQR 32). By comparison, the mean UK population health index and health state have been measured as 0.860 and 82.48 respectively [3]. The health indices and health states were higher for women than for men (Mann-Whitney *p*<0.001, *p*<0.001), and were highest in people with RRMS compared to other types of MS (Kruskal-Wallis *p*-<0.001, *p*<0.001). From previous studies, it is known that the distribution of types of MS differs between the genders [10], [17], but when gender was controlled for, health indices and health states were also highest in RRMS in both men and women (Kruskal-Wallis all *p*-values<0.001). Further details are given in Table 1, including values by gender and type of MS.

**Table 1:** Timelines, health indices and health states of the cohort.

Categories	N	Mean	SD	SE	Median	IQR	Range
**Age (yrs):**							
All	4506	50.7	11.2	0.17	51.0	16	20 to 87
Men	1301	52.8	11.4	0.31	53.0	16	23 to 87
Women	3197	49.9	11.4	0.20	50.0	16	20 to 84
**Time since diagnosis (yrs):**							
All	3575	10.9	8.8	0.15	9.0	12	0 to 63
Men	1023	11.3	8.9	0.28	10.0	13	0 to 48
Women	2535	10.8	8.9	0.18	9.0	12	0 to 63
**Health index (decimal)**							
All	4516	0.567	0.207	0.003	0.596	0.242	0.014 to 1.00
Male	1301	0.542	0.209	0.006	0.566	0.299	0.014 to 1.00
Female	3198	0.577	0.206	0.004	0.596	0.195	0.014 to 1.00
PPMS	654	0.496	0.183	0.007	0.511	0.295	0.014 to 1.00
RRMS	2747	0.595	0.209	0.007	0.596	0.227	0.075 to 1.00
SPMS	360	0.466	0.189	0.010	0.503	0.291	0.014 to 1.00
DKMS	661	0.577	0.198	0.008	0.596	0.219	0.014 to 1.00
**Health status (0 to 100)**							
All	4516	59.73	22.4	0.33	61.0	32	0 to 100
Men	1301	56.67	22.8	0.63	60.0	34	0 to 100
Women	3198	61.01	22.1	0.39	64.0	30	0 to 100
PPMS	654	54.84	22.2	0.87	59.0	30	0 to 100
RRMS	2747	62.02	22.9	0.42	64.0	30	0 to 100
SPMS	360	51.10	22.1	1.16	51.0	30	0 to 100
DKMS	661	59.89	22.9	0.89	60.0	35	0 to 100

Descriptions of the variables are shown. Slight differences in totals within categories compared to all are due to the small percentages (<2%) of participants for whom either age, gender or type of MS was missing.

**Figure 1:**
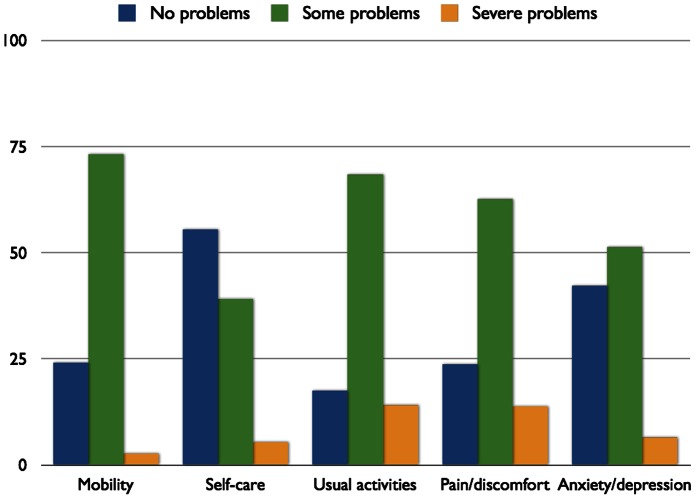
Health profiles of the cohort of people with MS. The frequencies of people with MS reporting no problems (level 1), some problems (level 2) and severe problems (level 3) on each of the 5 dimensions are indicated to show the health profiles of the cohort.

Health profiles were examined to assess the proportions of people with MS at each level on the five dimensions, and the percentages of people reporting at least some problems can be seen in Figure 1. Over four-fifths of the respondents (82.5%) reported experiencing problems in carrying out their Usual activities, and over three-quarters had problems Pain/discomfort (76.3%) and with Mobility (75.9%). When gender was included as a factor, it was found that men experienced problems more frequently than women in Mobility, Self-care and Usual Activities (all chi squared *p*-values<0.001), but there was no significant difference between the health profiles of men and women in Pain/discomfort and Anxiety/depression. Health profiles were assessed by respondent age in bands, and were found to differ on all 5 dimensions across the age ranges (chi-squared, all *p*-values<0.001) with greater proportions of people with problems in Mobility, Self-care and Usual activities as age increased. A similar pattern was observed in the general UK population, as would be expected, but problems were more frequent in people with MS across the age bands [12]. However, the profile pattern was more variable within the dimension of Pain/discomfort, and within Anxiety/depression the frequencies of people experiencing problems showed some decrease with age. Analysis of the health profiles by type of MS showed that problems were more frequent in the progressive types of MS than in RRMS for the dimensions of Mobility, Self-care, Usual Activities and Pain/discomfort (chi-squared, all *p*-values<0.001). There were differences in the health profiles of Anxiety/depression by type of MS but the pattern was more variable (*p* = 0.03). Health profiles by age band, gender and type of MS are shown in Table 2.

**Table 2:** Health profiles of the cohort.

Categories:	Dimension														
%	Mobility			Self-care			Usual activities			Pain/discomfort			Anxiety/Depression		
	1	2	3	1	2	3	1	2	3	1	2	3	1	2	3
All	24.1	73.2	2.7	55.5	39.1	5.4	17.5	68.4	14.1	23.7	62.6	13.8	42.2	51.3	6.5
**Gender:**															
Men	17.8	78.7	3.5	50.5	41.7	7.8	14.8	66.9	18.3	22.8	63.2	14.0	43.4	49.3	7.2
Women	26.8	70.8	2.3	57.7	38.0	4.3	18.6	69.0	12.4	23.7	62.6	13.7	41.7	52.1	6.3
**Age band:**															
< = 39 (yrs)	45.6	54.4	1.0	70.5	27.1	2.3	32.7	59.8	7.4	33.0	55.7	11.3	37.7	52.2	10.2
40–49	29.1	68.8	2.1	60.8	35.6	3.6	18.6	68.5	13.0	22.5	63.7	13.9	38.9	53.5	7.7
50–59	17.1	80.3	2.6	49.1	45.8	5.1	12.2	72.1	15.7	19.5	65.1	15.4	41.6	52.6	5.8
60–69	13.6	82.1	4.3	48.3	42.9	8.8	12.3	70.0	17.7	23.3	63.0	13.7	48.5	48.3	3.2
> = 70	6.9	86.2	6.9	38.5	45.4	16.1	10.9	67.2	21.8	26.4	63.8	9.8	60.3	35.6	4.0
**Type of MS:**															
PPMS	4.6	89.9	5.5	39.8	48.9	11.3	4.7	72.3	22.9	17.7	64.8	17.4	43.9	51.8	4.3
RRMS	31.1	67.4	1.6	61.8	34.7	3.5	22.1	67.4	10.5	26.1	61.9	12.0	41.5	51.9	6.6
SPMS	3.1	89.7	7.2	32.5	56.1	11.4	4.4	65.3	30.3	18.1	64.4	17.5	36.9	55.8	7.2
DKMS	25.9	72.5	1.7	57.5	39.0	3.5	17.9	71.0	11.2	23.4	62.2	14.4	45.7	45.8	8.5

The health profiles of the cohort by gender, age band and type of MS are shown (*N* = 4516).

### Relationships between Continuous Variables

Since health indices are calculated values based on responses on the 5 dimensions, and health states are based on a respondent’s general perception of their quality of life, the relationship between health indices and health states was assessed using Spearman’s rank correlation. A moderately strong positive relationship was found (*rho* = 0.61, *p*<0.001). Having observed that the frequencies of problems varied with age, the relationships between age, duration of MS, health index and health state were measured. Weak negative relationships were found: *rho* = −0.16 for age against health index (*p*<0.001) and *rho* = −0.11 for age against health state. (*p*<0.001). Similarly, duration of MS against health index and against health state yielded values of *rho* = −0.20 and *rho* = −0.14, respectively (*p*<0.001, *p*<0.001).

### Lifestyle Factors

It has been observed that quality of life (health index and health state) varies with lifestyle factors such as educational attainment, employment status and living arrangements [3 UK norms] and these factors were examined in our cohort of people with MS. Among our respondents the highest educational levels attained were: 25.0% secondary school, 31.8% higher education, and 43.2% occupational or other qualifications. Of these, the highest health indices and health states were among those with higher education (Kruskal-Wallis *p*<0.001, *p*<0.001), as observed in the general UK population [3 UK norms]. However, within types of MS, there was no significant difference across the educational levels in the health indices of people with SPMS, or in the health states of people with either PPMS or SPMS. For people of workforce age, 47.5% were in gainful employment and this group reported higher health indices and health states than those who were not working (Mann-Whitney *p*<0.001, *p*<0.001). This was also the case within each type of MS (all *p*-values<0.001). Within the potential workforce, 31.3% reported their employment status as sick/disabled and, as would be expected, this group reported lower health indices and health states than those who were not sick/disabled (Mann-Whitney *p*<0.001, *p*<0.001); the same was observed for each type of MS (all *p*-values<0.001). Approximately three quarters (75.6%) of our respondents were living as part of a couple and the remainder were not, and their health indices and health states were compared based on this criterion. There was no significant difference in the health indices, but people living as part of a couple reported a higher health state (Mann-Whitney *p* = 0.04). However, within types of MS, the difference in health states was only significant for people with DKMS (p<0.001). The living arrangements of the respondents were also (separately) categorised on the basis of whether they lived with dependent children (41.9%), with non-dependents (52.3%) or alone (5.8%). People living with dependents had the highest health indices (Kruskal-Wallis *p*<0.001) and although they had the highest health states, the differences were not significant. These analyses led to assessing what general and MS-related factors are associated with high and low health states for the cohort.

### Factors Associated with High and Low Health States

It had been observed that the UK general population mean health state (of 82.48) [3] was approximately 1SD above the cohort mean (of 59.73, SD 22.4). This was used as the basis for characterising a high health state group (High-VAS, having health states at least 1SD above the mean, *N* = 731) and a low health state group (Low-VAS, having health states at least 1SD below the mean, *N* = 695). Beginning with an analysis of factors associated with high health states, the general and MS-specific characteristics of the High-VAS group were compared with those with a health state below this value. It was found that the High-VAS group was characterised by better health profiles on all 5 dimensions (all chi squared *p*<0.001), higher health indices (Mann-Whitney *p*<0.001), younger age (Mann-Whitney *p*<0.001), shorter durations of MS (Mann-Whitney *p*<0.001), female gender (chi squared *p*<0.001), RRMS (chi squared *p*<0.001), higher educational attainment (chi squared *p*<0.001), and being in paid employment (chi squared *p*<0.001). A similar analysis comparing the Low-VAS group with the remainder of the cohort showed that the factors associated with the Low-VAS group were: poorer health profiles on all 5 dimensions (all chi squared *p*<0.001), lower health indices (Mann-Whitney *p*<0.001), older age (Mann-Whitney *p* = 0.0014), longer durations of MS (Mann-Whitney *p*<0.001), male gender (chi squared *p*<0.001), a progressive type of MS (chi squared *p*<0.001), lower educational attainment (chi squared *p*<0.001) and having an employment status of sick/disabled (chi squared *p*<0.001)). Particular living arrangements (as categorised earlier) were not associated with either the High-VAS or Low-VAS groups. Significance (at the 95% level) was retained for all variables when a Bonferroni correction was applied (based a required *p* value of 0.0042). Logistic regression was used to estimate which of the health profile dimensions were most likely to predict a High-VAS or Low-VAS health state. Odds ratios of being in the High-VAS group were calculated relative to having severe problems (profile = 3). Better health profiles gave increased odds of being in the High-VAS group on all the dimensions with the most predictive being Usual Activities (Odds ratios: 13.33 [CI 10.98, 15.87] for some problems compared to severe problems, and 41.67 [CI 25.64, 66.6] for no problems compared to severe problems). Odds ratios of being in the Low-VAS group were calculated relative to having no problems (profile = 1). Poorer health profiles gave increased odds of being in the Low-VAS group on all the dimensions with the most predictive being Mobility (Odds ratios: 5.41 [CI 3.9, 7.46] for some problems compared to no problems, and 43.48 [CI 27.03, 71.43] for severe problems compared to no problems). All the odds ratios and confidence intervals are compared in Table 3 (all *p*-values<0.001).

**Table 3:** Odds ratios for high and low health states.

Dimension	High-VAS		Low-VAS	
	Some problems: severe problems	No problems: severe problems	Some problems: no problems	Severe problems: no problems
Mobility	10.50 [8.77, 12.5]	23.80 [8.77, 66.67]	5.41 [3.9,7.46]	43.48 [27.03, 71.43]
Self-care	7.57 [4.10, 13.89]	10.99 [8.33, 14.49]	4.27 [3.52, 5.18]	13.70 [10.10, 18.52]
Usual activities	13.33 [10.98, 15.87]	41.67 [25.64, 66.67]	4.29 [2.87, 6.45]	22.22 [14.49, 33.33]
Pain/discomfort	5.99 [5.05, 7.14]	22.22 [13.89, 37.03]	2.44 [1.87, 3.18]	7.87 [5.88, 10.64]
Anxiety/depression	5.00 [4.15, 5.99]	11.49 [6.06, 21.73]	2.47 [2.03, 3.00]	8.77 [6.62, 11.63]

The odds ratios [and confidence intervals] of being in the High-VAS group or the Low-VAS group based on the level of problems reported on the 5 dimensions are shown.

## Discussion

### Main Findings

This large-scale study has used over 4500 responses to the EQ-5D to examine how people with MS rate their health-related quality of life by assessing the health profiles, health indices and health states of the cohort. Analysis of the health profiles revealed a high prevalence of problems in all 5 domains, with less than 25% of people reporting no problems with Mobility and with Pain/discomfort and less than 20% reporting no problems in carrying out their Usual Activities. As might be expected, there were differences in the patterns of problems between the genders and between people with different types of MS, with greater proportions of men and people with a progressive type of MS usually experiencing more problems. The frequencies of problems increased with age, as observed in the general UK population [3], but this was not the case with Anxiety/depression, where the converse was observed to some extent. This sort of pattern was also noted in a previous study where the HADS scores (particularly for anxiety) of people with MS decreased with age [10]. Considering that the health indices are calculated from the health profile scores, it is worth bearing in mind that a person’s health index measured repeatedly over the disease course may be comprised of health profiles varying in either direction, and it is possible that this effect could partly obscure health deteriorations in some dimensions. The health indices of the cohort were notably lower than those of the general UK population. In fact, a comparative study of well over a hundred health conditions found that the health indices of people with MS are among the very lowest [13].

There was a moderately strong positive relationship between health indices and health states. Unlike health indices, health states are based on the respondent’s overall feeling about their quality of life, and so are not based directly on clinical symptoms. In a study focussing on asthma, it was found that clinical status does not always predict self-rated health state [18]. This may be because people with a chronic condition develop coping strategies to realign their expectations and experience and so they may rate their quality of life higher than might be expected from their clinical status [19]. However, that should not be a reason to underestimate the impact of their condition on their well-being, but rather it shows how people are adapting to their circumstances [20].

In common with the general UK population, it was found that higher educational attainment was associated with higher health states in the cohort as a whole. However, this relationship did not hold for people with PPMS or SPMS, for whom there was no significant difference in health states by educational attainment and this may be due to the impact of their condition. Generally, there were no significant differences in the health states of people according to their living arrangements. As would be expected, people in paid employment reported higher health states than those who were not working, and a recent cross-sectional UK study has highlighted the association between detachment from the labour market and poor self-rated health [21]. The ability to remain active and to be able to take part in the workforce is an important factor that we highlighted in a previous study [11].

People with high health states (High-VAS) had different characteristics to the remainder of the cohort, and, of course, some of these factors are related; for example younger age and shorter duration of MS, being female and having RRMS. The people with low health states (Low-VAS) also differed from the remainder of the cohort and tended to have the opposite characteristics to the High-VAS group. As mentioned earlier, some of these factors were to be expected due to the nature of MS. But, it was not anticipated which (if any) of the EQ-5D dimensions would be most predictive of a high or a low health state. The comparison of the health profiles showed that being able to engage in Usual Activities was most predictive of being in the High-VAS group, whereas problems with Mobility were most predictive of a low health state (Low-VAS). Though all the dimensions were predictive of health state, a tabulation of the odds ratios (Table 3) showed that Anxiety/depression and Pain/discomfort were among the least predictive dimensions of being in either the High-VAS or Low-VAS group. There may be a variety of reasons for this: for example, it may be that problems in these dimensions can be managed by therapy and medication. In contrast, not being able to enjoy their Usual Activities (High-VAS group) and difficulties with Mobility (Low-VAS group) may be associated with irreversible deterioration in physical functions, and with loss of social contact and participation in general life activities. Even so, this does not detract from the considerable impact of pain and mental health issues on the lives of people with MS. High scores on all the dimensions were highly predictive of a low health status, and conversely, low scores were highly predictive of a high health status. The magnitude of these effects should be considered clinically relevant when caring for people with MS and seeking to promote their quality of life.

### What this Study Adds

This is the largest known study of health-related quality of life via the EQ-5D in people with MS that has been reported to date. It has shown the high prevalence of problems in all 5 dimensions of the health profile, and how they vary when different factors are taken into consideration. It has compared the health states of people with MS with those of the general UK population, and found them to be considerably lower, such that the mean health state for the general population is 1SD above the cohort mean. Having access to a large cohort has allowed robust sub-group analyses that would not have been possible with smaller sample groups. This included being able to divide the cohort into High-VAS and Low-VAS groups to assess the role of various factors in predicting a high or low health state. Other examples were being able to examine the influence of factors, such as educational status, or living arrangements, on quality of life for people with different types of MS. This study provides new insights into the patterns of factors that make up quality of life in people with MS and these highly significant findings should be considered clinically relevant to guide the provision of the best care for people with MS and to inform further research.

### Limitations

This study was conducted via the web portal of the UK MS Register and the respondents were self-selected, and so it is possible that the data may not be fully representative of the prevalent MS population. Self-selection may result in response bias and the use of web-based data collection methods may pose a barrier to some groups of people, such as the elderly, socially disadvantaged or the technically inexperienced [22], [23]. However, the value of remote data collection methods is increasingly being recognised in epidemiologic studies, with the evidence largely supporting comparability between web and mail as media for questionnaire delivery [24]. The educational attainment profile of our cohort is similar to that of the general UK population, as are many of the MS-specific characteristics, except that our proportion of people with SPMS is a little lower than expected [5]. This may be because of poorer health among people with SPMS or because of diagnosis changes, as people with SPMS would have firstly been diagnosed with RRMS, and they may be reporting their earlier diagnosis [4], [25]. We will be able to assess any bias in the portal data using data linkage as the clinical and routine data accrue. To do this we will anonymously-link the portal data, clinical and routine data and compare demographic factors (such as age and gender) and MS-specific factors (such as type and duration of MS) across the datasets. We will use a population administrative register as the gold standard for the demographic variables, and the clinical caseload as the gold standard for the MS-specific variables. We will then be able to quantify bias in the portal data and propose correction factors (where relevant) to be used in generalising findings to the MS population in general. The UK MS Register is still in its early stages, and although many studies will be made possible as the data increase over time, others will still require more traditional settings and data collection methods [26].

### Future Work

A programme of further work is underway to analyse additional questionnaires delivered via the portal. This will include studying quality of life (via the EQ-5D), mental health (via the HADS) and the physical and psychological impact of MS (via the MSIS-29) in relation to self-reported symptoms and medication reports. Through the increasing numbers of participants on the portal, and their periodic return to repeat the questionnaires, we will be able to explore additional methodologies and to carry out longitudinal studies. We plan to build upon our qualitative research and extend our engagement with people with MS, and other stakeholders, to ensure that the Register meets their needs and expectations [27]–[29]. As the data accrue we will be able to link clinical, routine and portal datasets to compare the information provided and to carry out additional studies, only possible via individual-level data linkage. This will include comparing the Expanded Disability Status Scale (EDSS) scores from clinical data with self-assessed outcome measures received via the portal, and factoring in self-reported symptoms, such as fatigue, which are known to be important to people with MS, but not captured directly by the EDSS or EQ-5D [30].

### Conclusion

The importance of quality of life cannot be over-estimated, and the EQ-5D is a standardised, health-related quality of life measure that is widely used to assess general populations and condition-specific cohorts [12], [13]. This large-scale study, with over 4500 participants, has used the EQ-5D to show how people with MS rate their health-related quality of life, and the factors that impact upon their health status. The findings can be used to inform care provision and further research to work towards enhancing the quality of life of people with MS.
